# Hotspots Reduction for GALS NoC Using a Low-Latency Multistage Packet Reordering Approach

**DOI:** 10.3390/mi14020444

**Published:** 2023-02-14

**Authors:** Zhenmin Li, Ruimin Shen, Maoxiang Yi, Yukun Song, Xiaolei Wang, Gaoming Du, Zhengfeng Huang

**Affiliations:** School of Microelectronics, Hefei University of Technology, Hefei 230009, China

**Keywords:** network-on-chip, packet reordering, hotspots reduction

## Abstract

Traffic splitting enabled by Globally Asynchronous Locally Synchronous (GALS) Network-on-chip (NoC) brings multipath routing capability, which significantly increases link bandwidth at the cost of out-of-order packet delivery. Solving the packet reordering problem is one of the keys to ensure the quality of service (QoS) for NoC. However, traditional packet reordering approaches rely on local reorder buffer, causing on-chip hotspots, which aggravates chip aging and even leads to interconnection failures. In this paper, we present a multistage packet reordering (MPR) approach, which cannot only reduce the transmission latency but also effectively reduces hotspots caused by local reordering. Specifically, we propose multistage reordering buffer (MRB) by reusing channel buffers for implementing MPR. Experimental results show that our proposed approach achieved improved thermal efficiency with reduced hardware resource consumption.

## 1. Introduction

**Figure 1:**
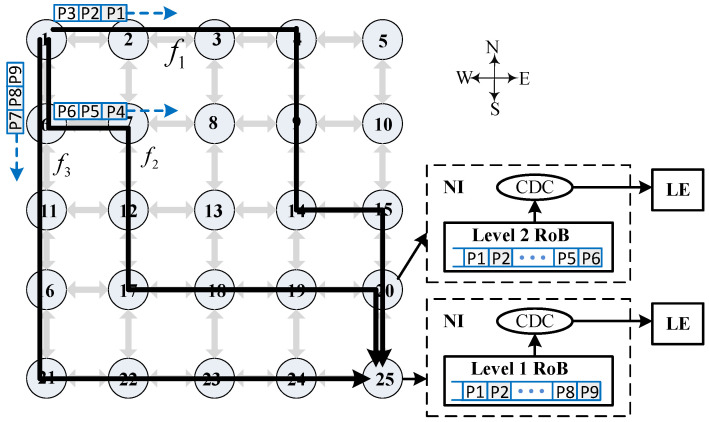
Illustrative example of GALS NoC with single- and multistage packet reordering scenarios.

On-chip communication has come to play a pivotal role in high-performance multiprocessor system-on-chip (MPSoC) design as technology geometries keep decreasing to deep submicron regime. Because of the superior regularity, scalability and performance, network-on-chip (NoC) has become the de facto solution for interconnecting hundreds of components, such as processor cores and memories [1]. Compared with bus-based or point-to-point communication schemes, higher link bandwidth-enabled multipath routing is one of the remarkable advantages of NoCs, which further facilitates load balance and resource utilization. The globally asynchronous, locally synchronous (GALS) scheme is known to be superior in dealing with the growing complexity of MPSoCs due to the mitigation of the clock distribution problem and reduction in the dynamic power consumptions [2]. Figure 1 depicts a typical GALS NoC architecture, featuring a two-dimensional mesh topology. The network interface connecting to the synchronous or mesochronous router contains clock domain crossing (CDC), and synchronous circuitry is required for providing GALS architecture. The local element linking to the NoC through network interface is typically a processor containing local memory. End-to-end communication is achieved by establishing paths of data packets through network interfaces and routers.

The majority of GALS NoC architectures adopt a packet-switched intracommunication approach. However, because of the increasing complexity of network congestion, the unbalanced path delay inevitably disrupts the order of the data packets arriving at the destination router, which is identified as the out-of-order problem [3]. Multipath routing of NoC brings significantly higher link bandwidth at the cost of out-of-order data packet delivery. In-order packet delivery is required by a number of real-world applications, such as multimedia or cache coherence protocols. Guaranteeing that the packets are transmitted in an orderly manner has become a pivotal research topic. Murali et al. [3] presented a flow control method to maintain delivery order. This approach suffers from traffic congestion exaggeration and low resource utilization. Du et al. [4] overcame it by proposing a network calculus model for determining worst-case reorder buffer size. However, these approaches perform reordering at the local network interface of the router, causing a large amount of data operations.

The traditional local reordering approach waits until all data packets have arrived and performs total ordering, which consumes both excessive hardware resource usage and reordering time. Longer transmission delay not only exacerbates system performance but may also cause heavier traffic congestion, probably resulting in the deterioration of thermal efficiency. The power density of an NoC router is typically higher than the average power density of the processing elements [5], and thus the traditional local reordering solution may cause severe thermal problems. For instance, Figure 1 presents an illustrative example for local reordering of data packets, where *f* denotes an infinite flow of unicast traffic sent from the source router 1 to the destination router 25. A multipath routing algorithm is employed, splitting *f* into three different subflows, namely $f_{1}$, $f_{2}$ and $f_{3}$. Let us assume that the package IDs are smaller in $f_{1}$, medium in $f_{2}$, and larger in $f_{3}$. We further assume an unbalanced network congestion, resulting in out-of-order delivery of data packets. Traditional approaches perform reordering of packets at router 25, utilizing a reorder buffer (RoB) as part of the network interface.

Thermal issues have become one of the dominant factors that debase the reliability and performance of NoCs. Thermal hotspots with high temperature decelerate circuit switching, enlarge leakage power and increase system vulnerability [6]. In extreme cases, hotspots can even cause physical damage of the circuit and bring system failure. Due to high power density, NoC routers are one of the most influential sources of thermal hotspots [7]. Eliminating or alleviating hotspot issues has become a major design concern for NoCs. Li et al. [5] proposed an RoB-Router containing RoBs in virtual channels to mitigate head-of-line (HoL) blocking, reduce the conflicts in switch allocation and improve NoC performance. However, the RoB-Router cannot avoid local reordering of data packets at the network interface of the destination router. Moreover, the linked list data structure of the RoB-Router causes large maintenance overhead, giving rise to low cycle efficiency. Inspired by the fact that subflows produced by traffic splitting inevitably merge before final destination router, the time consumed by local reordering can be shortened if we can perform partial ordering of the data packets at these converging points. We can advisedly perform data packet reordering at the converging point of subflows, namely converging routers, to achieve better thermal equilibrium and mitigate the thermal hotspots caused by local reordering.

In this paper, we propose a multistage packet reordering approach utilizing a novel multistage reordering buffer, which can effectively reduce hotspots caused by local packet reordering. To the best of our knowledge, the proposed approach is the first attempt to alleviate the hotspot problem for GALS NoC using multistage data packet reordering while consuming reasonable hardware resources through channel buffer reuse. Our major contributions can be summarized as follows:

- We propose a multistage packet reordering (MPR) approach for GALS NoC to mitigate the hotspot issue. Data packets are reordered at designated converging points of subflows. MPR effectively mitigates thermal hotspots caused by local reordering and thereby improves the thermal safety and performance of NoC.
- We design a multistage reordering buffer (MRB) by reusing channel buffers for implementing MPR. MRB features an elastic dual-area (FIFO area and RoB area) buffer structure with a configurable packet size and burst length of target flow. MRB enables multistage reordering while minimizing resource usage.
- We extend the thermal model to measure energy consumption of NoC data transmission, including packet reordering. The proposed approach is implemented in FPGA and evaluated using both synthetic and industrial use cases. Experimental results show that our approach is significantly improved in both thermal efficiency and hardware resource usage.

This rest of this paper is organized as follows. Section 2 presents related work. Section 3 introduces the thermal model employed to measure NoC temperature distribution. Section 4 describes the multistage reordering approach in detail. The experimental results are given and discussed in Section 5. Finally, we conclude our paper in Section 6.

## 2. Related Work

A series of studies have been conducted on the multipath routing of NoC. The unbalanced workload distribution and routing algorithm can cause the hotspots, which can lead to the shorter lifetime of NoC. Ronhbani et al. [8] proposed a location-based aging elastic Xy-Yx routing algorithm to improve the chip lifetime. Wang et al. [9] used a nonminimal routing scheme to detour the traffic away to avoid hotspots. Chao et al. [10] proposed a routing-based traffic migration vertical-downward lateral-adaptive proactive routing algorithm to achieve load balancing and temperature balancing.

Since real-time systems require predictable time platforms for static analysis of the worst-case time, Martinet al. [11] proposed a time-predictable NoC multicore architecture for embedded systems designed for the worst case scenario. For the NoC worst performance analysis, Du et al. [12] made a preliminary attempt on the worst-case performance analysis of multipath minimal routing 2D-NoC. Du et al. [13] have put forward a heuristic method to minimize the worst-case delay bound of the target flow for multipath routing NoC. A number of NoC architectures proposed support the GALS scheme [14,15,16,17], as well as featuring clocking schemes such as mesochronous [14], asynchronous [15], or source synchronous [16]. Evangelia et al. [15] proposed an on-chip network architecture for global asynchronous and local synchronization, which efficiently controls routers by means of time-division multiplexing to improve the NoC performance.

Due to the inconsistency of network delay in multipath routing, the out-of-order situation of packets arriving at the destination necessitates the reordering of the received packets. Ebrahimi et al. [18] presented a dynamic buffer allocation structure to improve overall NoC performance. Daneshtalab et al. [19] further extended this work and proposed a simplified adaptive reorder mechanism which can dynamically adjust buffer allocations to improve resource utilization. Kwon et al. [20] proposed a reorder approach using an in-network reorder buffer, so as to ameliorate the utilization of the reorder buffer resource. It is of great significance to economize on reorder buffer, since they are expensive in terms of both resource and power consumption. However, the aforementioned approaches did not solve the thermal issue brought by local reordering strategy for data packets reordering.

## 3. Preliminary

In this section, we firstly introduce the basic thermal model, then extend this model to measure energy consumption of NoC data transmission, including packet reordering. After that, we introduce the target NoC platform upon which we propose our MPR approach. This platform is general enough to be extended to a wide range of NoCs.

### 3.1. Thermal Model of NoC for Temperature Calculation

The power consumption fluctuation decides the temperature variation of NoCs, which is crucial for constructing the thermal model. In this paper, the calculation of temperature distribution of NoC is based on the NoC thermal model presented in [21]. The overall temperature is decided by the energy consumption of both computation and communication. We focus on the energy consumption of communication, as the energy consumption of computation is determined by the characteristics of local processing cores, storage elements, or I/O peripherals. The energy consumption generated by unit data communication is calculated by the Formula (1).

(1) $$E_{bit}=E_{Rbit}+E_{Lbit},$$

where $E_{Rbit}$ denotes the energy consumption by the unit data communication for a router, and $E_{Lbit}$ is the energy consumption of the link between any two adjacent routers generated by the unit data communication. $E_{Rbit}$ can be further calculated by the Formula (2).

(2) $$E_{Rbit}=E_{Sbit}+E_{Bbit}+E_{Wbit},$$

where $E_{Sbit}$, $E_{Bbit}$ and $E_{Wbit}$ are the energy consumptions of switch, cache and internal connection, respectively. The energy consumption of router $P_{R}$ can be calculated using the Formula (3).

(3) $$P_{R}=E_{Rbit}\times B,$$

where *B* is the router bandwidth.

According to Fourier thermal theory, the heat flow is represented by electric current; temperature is represented by voltage; thermal conductance is denoted by conductance; and heat capacity is characterized by capacitance [22]. Therefore, the temperature of a router in NoC can be determined using the Formula (4).

(4) $$T=A^{-1}\times P,$$

where *A* is thermal conductivity matrix with the dimension of n×n. $A^{-1}$ is the thermal impact matrix, which can be described using the Formula (5).

(5) $$A=\left[\begin{array}{cccc} G_{1} & G_{2} & G_{3} & 0\\ G_{2} & G_{1} & 0 & G_{3}\\ G_{3} & 0 & G_{4} & G_{2}\\ 0 & G_{3} & G_{2} & G_{4} \end{array}\right],$$

where $G_{1}=g_{inter}+g_{intra}$. $G_{2}=-g_{intra}$. $G_{3}=-g_{inter}$. $G_{4}=g_{hs}+g_{inter}+g_{intra}$. Specifically, $g_{intra}$ is intralayer thermal conductivity, $g_{inter}$ is interlayer thermal conductivity and $g_{hs}$ is radiator thermal conductivity.

According to Fourier thermal theory, *P* represents the energy consumption matrix, which is given by the Formula (6).

(6) $$P=P_{n\times 1}+G_{5}S_{n\times 1},$$

where $P_{n\times 1}$ represents the bandwidth matrix of NoC, and $S_{n\times 1}$ represents the unit matrix. $G_{5}$ is determined by $g_{hs}\times T_{amb}$, where $T_{amb}$ is the ambient temperature.

We extend the current thermal model to measure the energy consumption of NoC data transmission, including packet reordering, as shown in the Formula (7).

(7) $$P=\left(P_{n\times 1}+Q_{n\times 1}\right)+G_{5}S_{n\times 1},$$

where, $Q_{n\times 1}$ represents the reorder buffer matrix. In this paper, we focus on the two-dimensional NoC topology, while Formula (4) is general enough to be applied to NoCs with various topologies.

### 3.2. Target NoC Platform with Reorder Buffers

**Figure 2:**
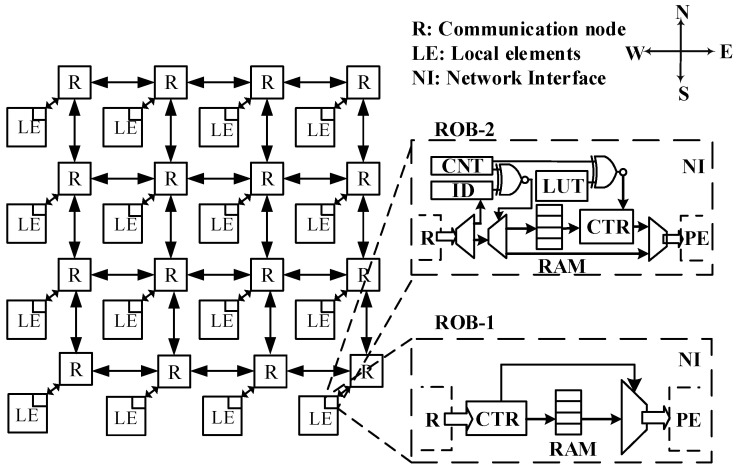
Architecture of the target 2D mesh GALS NoC platform with different types of reorder buffers (RoB-1 and RoB-2) for single-stage packet reordering.

The GALS NoC communication infrastructure employed in this paper features an expandable mesh topology [23], as shown in Figure 2. Each node in this NoC architecture contains a set of synchronous router, network interface and local element. The local element can be a processing core, storage element or I/O peripheral connected with the NoC through network interface. The wormhole switching strategy is adopted for NoC routers. For the sake of simplicity, network interfaces are not depicted in Figure 2. The NoC routers perform data packets buffering and forwarding according to the established routing algorithm. We assume the classical XY routing algorithm, while our proposed MPR approach is independent from a specific routing algorithm. Moreover, we assume that the NoC routers are implemented without virtual channels, since we are targeting the packet reordering issue caused by traffic splitting rather than the HoL problem.

In a traditional packet reordering scheme, an RoB module is located within the network interface of each router, which is responsible for reordering data packets in an orderly manner before forwarding them to LEs. We refer to this strategy as the single-stage reordering approach. A trivial implementation of the RoB is depicted as RoB-1 in Figure 2. RoB-1 mainly consists of a RAM block and a control logic. The out-of-order data packets are stored in the designated address according to their packet IDs. The entire target flow is forwarded to the local element only after all the packets arrive, thus guaranteeing in-order delivery. However, RoB-1 demands excessive hardware resource consumption, companioned by high power consumption and more hotspots due to a lot of local data reads and writes, especially when the target flow comprises a large amount of packets.

Depending on the requirement of input data imposed by the local element, the forwarding strategy of data packets can be categorized into two classes: (1) all data packets of the target flow must be reordered before being forwarded to the local element, and (2) a data packet is forwarded as long as it is in the correct order. For the latter case, an improved version of RoB-1, named RoB-2, is shown in Figure 2. The ID of a data packet is compared with a counter whenever it arrives. The packet is directly forwarded if its ID equals the counter value; otherwise, it is stored into a RAM block with the packet index updated into a look-up table (LUT). Whenever a packet is forwarded to the local element, the packet with the next ID is searched within the LUT. The transmission latency of packets of target flow is significantly reduced by RoB-2 structure. The multistage reordering approach, as well as the design of the multistage reorder buffer, is described in detail in the following section.

## 4. Multistage Reordering Approach

In this section, we present the design of MRB as a enabling component for achieving low-latency multistage packet reordering. Moreover, we describe the procedure of packet reordering using MRB using pseudocode. As illustrated in Figure 1, we can perform reordering of packets at each converging node. The number of converging points is bounded by

(8) $$N_{msr}=M_{s}-1,$$

where $N_{msr}$ is the number of routers eligible for performing MPR and $M_{s}$ is the maximum number of subflows forked along the transmission path.

### 4.1. Architecture of Multistage Reorder Buffer

**Figure 3:**
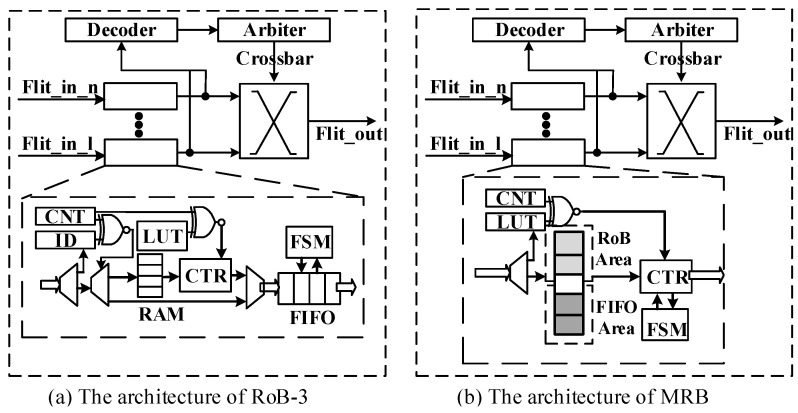
The architecture of NoC router with RoB-3 and MRB for multistage packet reordering.

The NoC router is responsible for receiving and forwarding the data packet. As shown in Figure 3, each router consists of a set of input channels, together with a decoding module, an arbitration module and a crossbar module, responsible for parsing the packets, making decisions of switching and establishing the forwarding path, respectively.

The traditional local reordering strategy can only support single-stage packet reordering, and the data packets that have been sorted will not be sent back into the on-chip network again. Therefore, RoB-1 and RoB-2 can no longer suffice for MPR. A straightforward solution is to instantiate an RoB similar to RoB-2 in each input channel of the NoC router, connecting to the channel buffer, as shown in Figure 3a. We name this RoB architecture RoB-3. RoB-3 is capable of performing MPR but suffers from the following drawbacks: (1) excessive hardware resource consumption since the RAM block inside RoB and the channel buffer coexist in the router, (2) high transmission latency of packets due to series connection of the reorder buffer and channel buffer and (3) only has support for fixed burst length. To overcome the aforementioned shortcomings, we propose MRB, as shown in Figure 3b.

In order to improve the hardware usage efficiency, it is efficient to reuse the channel buffer for packets reordering. Based on RoB-3, we remove the channel buffer implemented as an FIFO and mark off two areas in the RAM block as the RoB and FIFO area. The RoB area is used to perform packet reordering, while the FIFO area is dedicated to holding the packets that are ready for switch allocation. The effectiveness of multistage packet reordering is twofold. Firstly, waiting time is probably inevitable for data packet forwarding in the case of traffic congestion. Multistage packet reordering can save part of the waiting time by performing partial reordering before data packet forwarding, resulting in shortened total reordering time. Secondly, the partial reordering of multiple converging points may occur concurrently, which further reduces the total reordering time. The depth of the original channel buffer has to be increase to avoid overflow.

**Figure 4:**
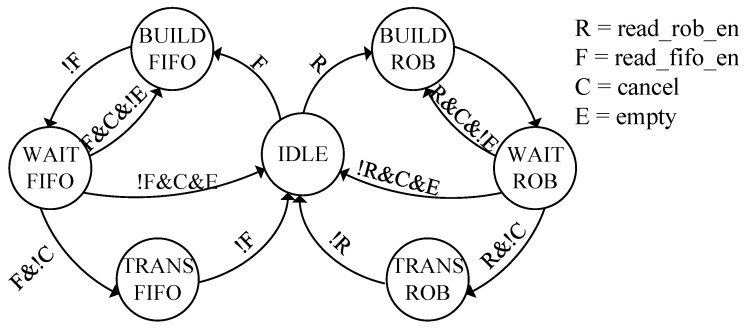
Diagram of finite-state machine controlling packet forwarding for multistage packet reordering using MRB.

The boundary of two areas, namely the RoB and FIFO area, is indicated by a pointer, which enables elastic operation of both areas. The fundamental operation of a router containing the proposed MRB in each input channel is controlled by a finite-state machine (FSM), as shown in Figure 4. The initial state of the FSM is named IDLE. From the IDLE state, the FSM transits to the BUILD-RoB state upon the arrival of a data packet of the target flow, and a request signal is sent to the decoder and the crossbar. Then, it enters the WAIT-RoB state by withdrawing the request signal for the decoder and maintaining the request signal for the crossbar. When the data packet is in the correct order, it is forwarded in the TRANS-RoB state, while the request signal for the crossbar is still asserted. The FSM is initialized to the IDLE state after the data packet is forwarded. When a data packet that does not require reordering arrives, the FSM changes to the BUILD-FIFO state. The WAIT-FIFO and TRANS-FIFO states behave similarly to the WAIT-RoB and TRANS-RoB state, respectively, except for that they perform only buffering before packet forwarding instead of reordering.

### 4.2. Packet Reordering Procedure Using MRB

The packet reordering procedure using MRB is described in Algorithm 1, which is implemented in the hardware of MRB. The procedure Packet_Input and Packet_Output is executed in parallel using a dual-ported RAM block. We use semaphores for mutual exclusive operation to the MRB. The size of the FIFO area is always more than the length of a unit burst to avoid MRB overflow. Referring to Algorithm 1, procedure Packet_Input (lines 1–9) places the packet into the correct area, while procedure Packet_Output (lines 10–20) performs packet reordering with the configurable burst length.
**Algorithm 1** The packet reordering procedure using multistage reorder buffer.**Intput:** Data packets sent from the previous NoC router, RoB area (*rob[i]*), FIFO area (*fifo[j]*), look-up table (*lut[i]*), burst length (*burst_length*) and flow length (*flow_length*).**Output:** 
Data packets in correct order and ready to be allocated for switching.1:**procedure** Packet_Input2:    **if** the packet arrived needs to be reordered **then**3:        rob[pt_empty]=data_in;4:        lut[pack_id]=pt_empty;5:        update *pt_empty* and look-up table;6:    **else**7:        fifo[cnt_fifo]=data_in;8:    **end if**9:**end procedure**10:**procedure** Packet_Output11:    **while** seq_num<flow_length **do**12:        **while** cnt_burst<burst_length **do**13:           **while** lut[cnt]==0 **do**14:               wait for the packet with the current packet ID number;15:           **end while**16:           cnt_burst++, seq_num++;17:        **end while**18:        obtain the address of packets having ID of range [seq_num−burst_length−1,seq_num−1] from look-up table and move these packets into FIFO area;19:    **end while**20:**end procedure**

## 5. Experiments and Results

In this section, we evaluate the proposed MPR approach in a range of metrics for synthetic benchmark and industrial patterns, including MWD and VOPD [24]. Specifically, we use Verilog hardware description language to design the target NoC platform containing four different types of reorder buffers, namely RoB-1, RoB-2, RoB-3 and MRB. The target NoC system features a 5×5 mesh topology. We set the data width to be 64 bits and the depth of buffer to be 64. The leaky bucket injection policy is enforced for data packet sending [25]. The injection rate *p* is set to 0.1, and the burst length *b* is set to 10 for all the experiments.

### 5.1. Hardware Resource Usage Comparison

We implemented the target NoC system containing four different types of reorder buffers presented in Section 3 and Section 4 using Xilinx XC6VLX760 FPGA. The synthesis results of a single NoC router using RoB-1, RoB-2, RoB-3 and MRB, respectively, in the target platform are shown in Table 1. Note here RoB-1 and RoB-2 are instantiated only once within the network interface of each router, while RoB-3 and MRB reside in every input channel of a router. As seen from the table, MRB achieves the lowest resource consumption. Compared with RoB-3, MRB consumes 22.0% less registers and 31.2% less LUTs, attributable to the reuse of channel buffers. The resource usage of MRB is comparable to RoB-1 and RoB-2, however, neither RoB-1 nor RoB-2 can support multistage reordering. In terms of operating frequency, the MRB achieves the highest among these four solutions: 47.6% higher than RoB-3. This improvement is because of the notable reduction in critical path enabled by channel buffer reuse.

### 5.2. Packet Transmission Delay Comparison

**Figure 5:**
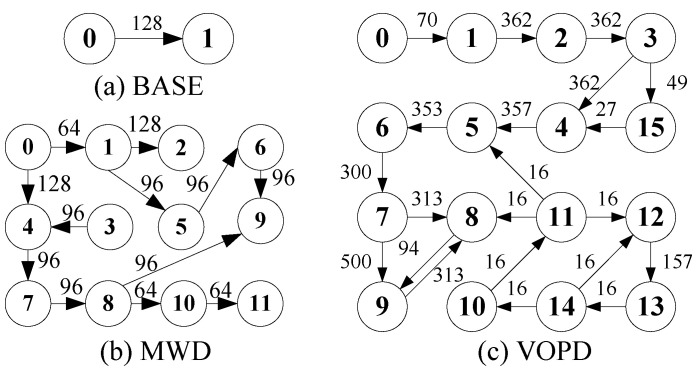
The task graphs of the BASE, MWD and VOPD benchmarks. The number in the circle represents node ID. The number beside the arrow represents the amount of communication between the two tasks.

We use industrial patterns MWD and VOPD, together with a synthetic benchmark BASE, to evaluate the packet transmission delay using four reorder buffer solutions used in the previous subsection. The task graphs of BASE, MWD and VOPD are shown in Figure 5. The arrows between two tasks represent data communication, with the number of data packets annotated on the arrows. Each data packet has 62 bits, consisting of five areas. The flit-type area has two bits, denoting the type of data flit. The sequence area has six bits, representing the sequence of flit. The packet ID area also has six bits, containing the packet ID. The flow ID area has 24 bits, showing the ID of the sending flow. The data area also has 24 bits, holding the payload of the data packet.

For the BASE, MWD and VOPD benchmarks, we map task 0 and 1 to router 1 and 25 in our 5×5 target NoC platform and randomly map other tasks. We set flow f(1,25) to be the target flow and all other flows to be contention flow. We evenly split the target flow into four subflows, which leads to three stages of packet reordering. For RoB-1 and RoB-2, a single stage of packet reordering at router 25 is implemented. We also added nine contention data flows to emulate a real-world NoC traffic scenario, and all the contention flows are split evenly at each router along their paths. The settings of these nine contention data flows are given in Table 2.

**Figure 6:**
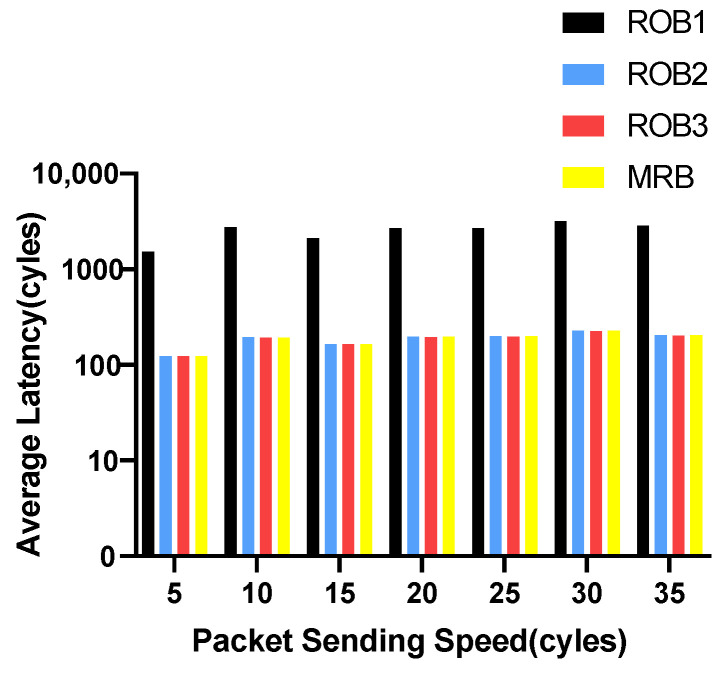
Average latency in varying packet sending speeding for VOPD.

**Figure 7:**
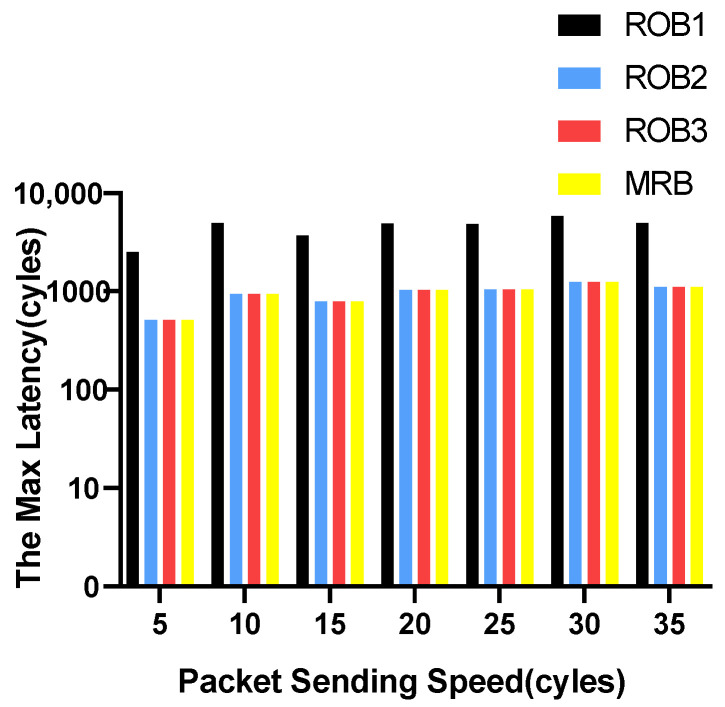
Maximum latency in varying packet sending speeding for VOPD.

**Figure 8:**
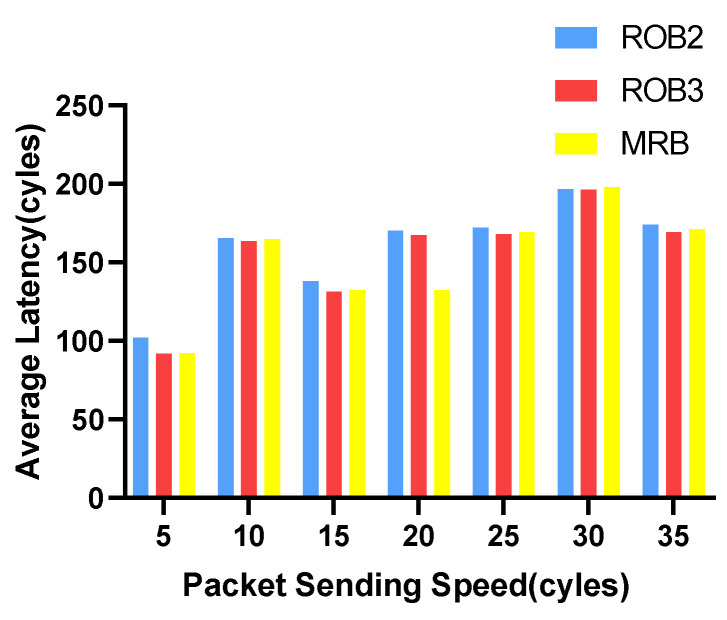
Average latency in varying packet sending speeding for MWD.

**Figure 9:**
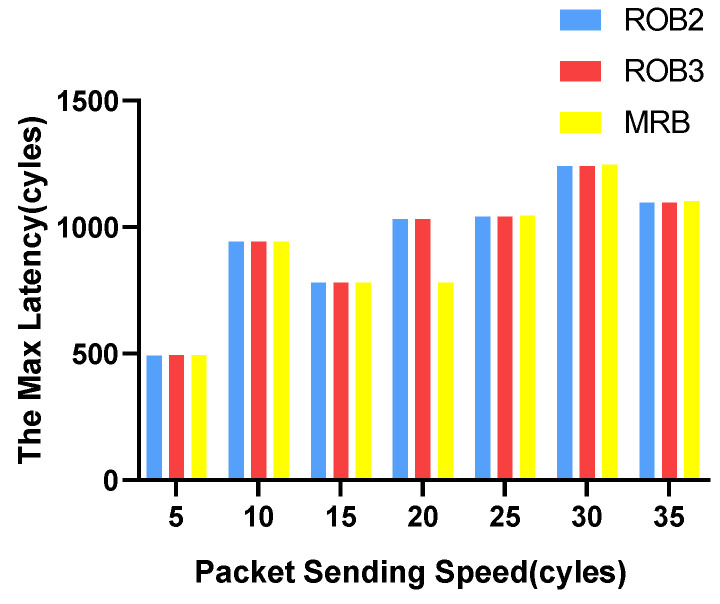
Maximum latency in varying packet sending speeding for MWD.

We vary the packet sending speed and measure the maximum and average transmission latency of data packets. The experimental results for VOPD are shown in Figure 6 and Figure 7. RoB-1 has the worst average and maximum latency, approximately ten times more than the other three solutions. The latency for RoB-2, RoB-3 and MRB are similar. The results for MWD are shown in Figure 8 and Figure 9. We remove RoB-1 from the benchmarks. For MWD, our MRB achieves the best average and maximum latency, especially at the sending speed of 20.

### 5.3. Thermal Efficiency Comparison

**Figure 10:**
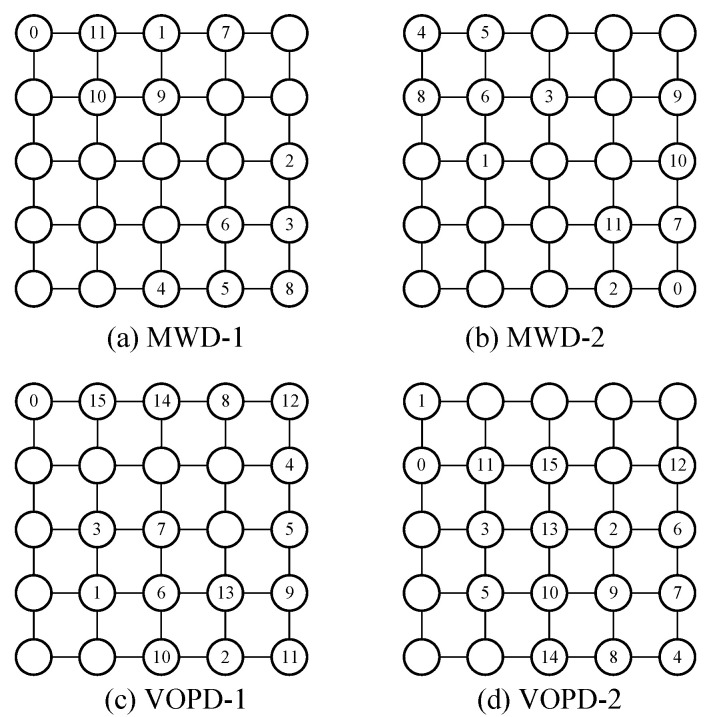
The mappings of task graphs for the MWD-1, MWD-2, VOPD-1 and VOPD-2 benchmarks.

**Figure 11:**
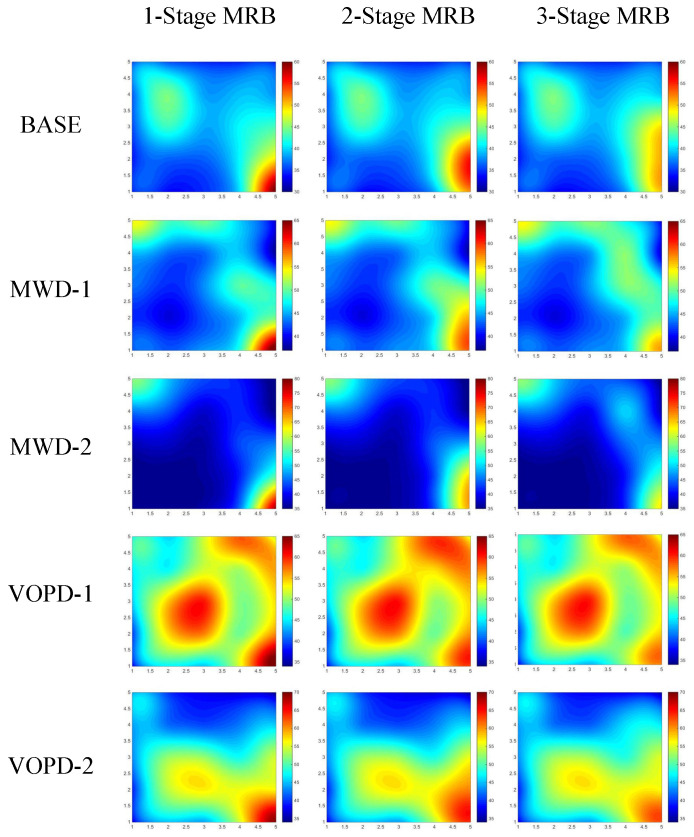
Thermal maps of benchmarks in different packet reordering scenarios.

In order to evaluate the thermal efficiency of the proposed MPR approach, we use the BASE benchmark together with industrial patterns MWD and VOPD to conduct the experiments. For the BASE benchmark, we map task 0 and 1 to router 7 and 25 in our 5×5 target NoC platform, referring to Figure 1. For MWD and VOPD, we use two different task mappings for each benchmark, respectively, named MWD-1, MWD-2, VOPD-1 and VOPD-2. These mappings of task graphs are shown in Figure 10. The thermal maps of our target NoC platform in different packet reordering scenarios are shown in Figure 11. We employ the thermal model described in Section 3.1 to calculate the temperature for each router and depict the thermal maps. The parameter setting of the thermal model is shown in Table 3.

In order to evaluate the thermal efficiency for different MPR settings, we set up three types of reordering scenarios, namely 1-Stage MRB, 2-Stage MRB and 3-Stage MRB. The target and contention flow settings are the same as those in Section 5.2. The target flow is split into four subflows, allowing a maximum of three stages of packet reordering. For scenarios 1-Stage MRB, 2-Stage MRB and 3-Stage MRB, we perform packet reordering in single-stage, two-stage and three-stage, respectively. The highest temperature values of all thermal maps depicted in Figure 11 are shown in Table 4. As seen from the table, 1-Stage MRB is practically the traditional packet reordering strategy. Compared with 1-Stage MRB, our MPR achieved 14.3% hotspots reduction on average. For each benchmark, the highest temperature keeps dropping as the stage of packet reordering increases. The largest improvement occurs for MWD-2, where the highest temperature is reduced by 18.9% using three stages of packet reordering. The experiments in this subsection effectively verify the hotspots reduction capability of our proposed MPR approach.

## 6. Conclusions and Future Work

In this work, we present a novel multistage packet reordering approach for GALS NoC, which cannot only reduce the transmission latency but also effectively reduces hotspots caused by local reordering. We propose a multistage reordering buffer by reusing channel buffers. In addition, we extend the thermal model to measure the energy consumption of NoC data transmission, including packet reordering. Experimental results show that our proposed approach achieved low transmission latency and improved thermal efficiency with smaller hardware resource consumption. We intend to explore the design space to further improve the efficiency of the multistage packet reordering technique. Moreover, the virtual channel will also be incorporated in the future work, as it is not currently supported by the MRB approach.

## Figures and Tables

**Table 1:** The FPGA synthesis results of NoC router using different reorder buffers.

	RoB-1	RoB-2	RoB-3	MRB
Registers	28,408 (100%)	30,172 (106.2%)	36,136 (127.2%)	28,184 (99.2%)
LUTs	47,735 (100%)	51,556 (108.0%)	65,308 (136.8%)	44,964 (94.2%)
Freq (MHz)	235.347	235.347	196.183	289.524

**Table 2:** The Contention Flow Settings.

Contention Flow ID	Source Node	Destination Node	Data Packet No.
Flow 1	(1, 2)	(4, 3)	985
Flow 2	(1, 4)	(5, 3)	530
Flow 3	(3, 1)	(4, 5)	664
Flow 4	(4, 2)	(1, 5)	778
Flow 5	(2, 2)	(5, 4)	184
Flow 6	(3, 3)	(4, 6)	360
Flow 7	(1, 3)	(6, 6)	148
Flow 8	(4, 1)	(2, 5)	896
Flow 9	(2, 1)	(4, 4)	746

**Table 3:** Parameters of the thermal model used for temperature calculation.

Parameter Name	Value
Router energy consumption	0.7066 nJ/bit
Router size	0.4 mm× 0.4 mm× 0.15 mm
Router thermal conductivity	100 W/(m× K)
Interface material size	0.00002 m (thickness)
Material thermal conductivity	4 W/(m× K)
Convective thermal resistance	0.1 K/W
Ambient temperature	25 (°C)

**Table 4:** The highest temperature of hotspots in thermal maps shown in Figure 11.

	BASE	MWD-1	MWD-2	VOPD-1	VOPD-2
1-Stage MRB	60.29 °C	66.50 °C	78.43 °C	65.70 °C	69.08 °C
2-Stage MRB	51.39 °C	58.59 °C	66.57 °C	60.11 °C	63.48 °C
3-Stage MRB	49.88 °C	56.63 °C	63.64 °C	58.73 °C	62.10 °C
Reduction	17.27%	14.8%	18.9%	10.6%	10.1%

## Data Availability

The data that support the findings of this study are available from the corresponding author upon reasonable request.
